# Use of Video Decision Aids to Promote Advance Care Planning in Hilo, Hawai‘i

**DOI:** 10.1007/s11606-016-3730-2

**Published:** 2016-05-18

**Authors:** Angelo E. Volandes, Michael K. Paasche-Orlow, Aretha Delight Davis, Robert Eubanks, Areej El-Jawahri, Rae Seitz

**Affiliations:** 1 Massachusetts General Hospital, 50 Staniford Street, 9th Floor, Boston, MA 02115 USA; 2 Harvard Medical School, Boston, MA USA; 3 ACP Decisions Nonprofit Foundation, Waban, MA USA; 4 Boston University School of Medicine, Boston, MA USA; 5 Hawai‘i Medical Service Association, Honolulu, HI USA

## Abstract

**Introduction:**

Advance care planning (ACP) seeks to promote care delivery that is concordant with patients’ informed wishes. Scalability and cost may be barriers to widespread ACP, and video decision aids may help address such barriers.

**Aim:**

Our primary hypothesis was that ACP documentation would increase in Hilo after ACP video implementation. Secondary hypotheses included increased use of hospice, fewer deaths in the hospital, and decreased costs in the last month of life.

**Setting:**

The city of Hilo in Hawai‘i (population 43,263), which is served by one 276-bed hospital (Hilo Medical Center), one hospice (the Hospice of Hilo), and 30 primary care physicians.

**Program Description:**

The intervention consisted of a single, 1- to 4-h training and access to a suite of ACP video decision aids.

**Program Evaluation:**

Prior to implementation, the rate of ACP documentation for hospitalized patients with late-stage disease was 3.2 % (11/346). After the intervention, ACP documentation was 39.9 % (1,107/2,773) (*P* < 0.001). Primary care providers in the intervention had an ACP completion rate for patients over 75 years of 37.0 % (1,437/3,888) compared to control providers, who had an average of 25.6 % (10,760/42,099) (*P* < 0.001). The rate of discharge from hospital to hospice for patients with late-stage disease was 5.7 % prior to the intervention and 13.8 % after the intervention (*P* < 0.001). The average total insurance cost for the last month of life among Hilo patients was $3,458 (95 % CI $3,051 to 3,865) lower per patient after the intervention when compared to the control region.

**Discussion:**

Implementing ACP video decision aids was associated with improved ACP documentation, greater use of hospice, and decreased costs. Decision aids that promote ACP offer a scalable and cost-efficient medium to place patients at the center of their care.

**Electronic supplementary material:**

The online version of this article (doi:10.1007/s11606-016-3730-2) contains supplementary material, which is available to authorized users.

## INTRODUCTION

In 2014, the Institute of Medicine (IOM) released a report stating that end-of-life care in America is broken.^1^ The report highlighted one potential solution: advance care planning (ACP), a shared decision-making approach that seeks to promote care delivery that is concordant with patients’ informed wishes.^1^ Subsequently, there has been a surge of activity surrounding ACP, with new regulations for reimbursement by the Centers for Medicare and Medicaid Services (CMS)^2^ and attention by the lay press.^3^,^4^

Two central barriers to widespread adoption of ACP have been scalability and cost.^1^ Over the last decade, our group, the Video Images of Disease for Ethical Outcomes (VIDEO) Consortium, has conducted controlled trials showing that ACP video decision aids promote more informed decision-making when supplementing more traditional modes of ACP.^5^–^15^ Although these video tools were highlighted in the IOM report as potentially effective and cost-efficient catalysts to ACP discussions,^1^ a large-scale translational trial has yet to be described in which the videos are integrated into clinical care.^16^–^18^

In late 2012, the Hawai‘i Medical Service Association (HMSA), an Independent Licensee of the Blue Cross and Blue Shield Association and a nonprofit mutual benefit society, sought to improve ACP rates statewide through innovative collaborations. Sponsoring a multi-year initiative, HMSA has made available a suite of evidence-based ACP video decision aids throughout the state at no cost to providers or patients, and without regard for insurance carrier or status (Appendix A, available online); we began this implementation as a collaborative effort between HMSA and our research group in early 2013.

With the help of community-based collaborations, we developed videos in Ilocano, Tagalog, Japanese, Cantonese, Vietnamese, Samoan, Korean, and Marshallese, languages commonly spoken in Hawai‘i. In the first year, we focused our efforts on Hilo, the most populous city on the Island of Hawai‘i. Our primary hypothesis was that ACP documentation would increase in Hilo after ACP video implementation. Secondary hypotheses included increased use of hospice, fewer deaths in the hospital, and decreased costs in the last month of life.

## METHODS

### Setting and Participants

**Fig. 1:**
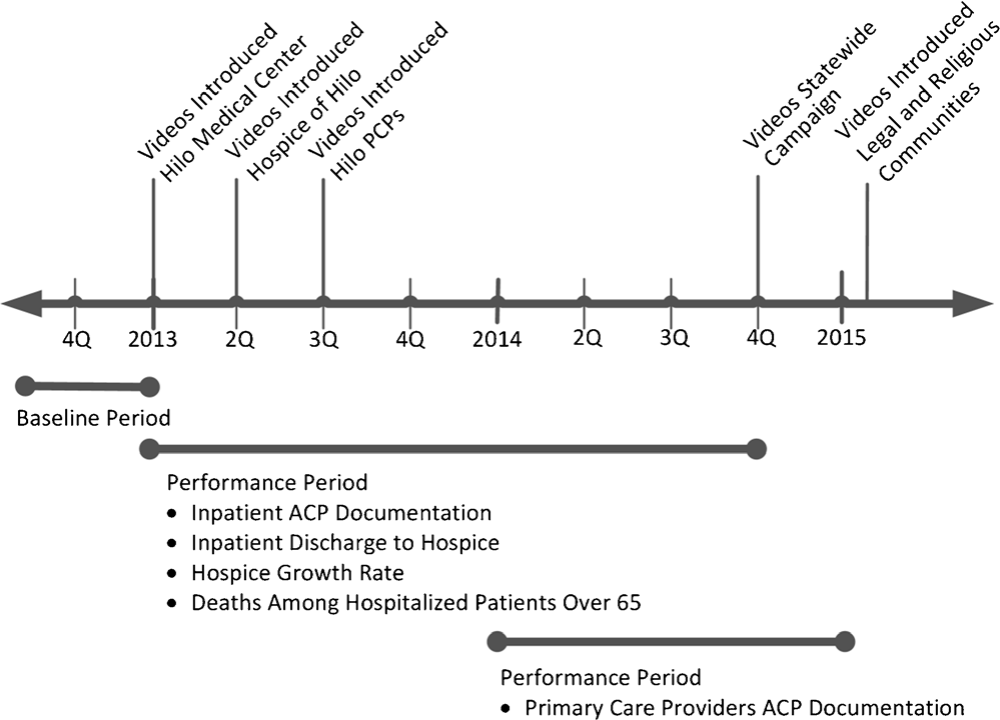
Timeline of the intervention

Hilo is home to 43,263 people from diverse backgrounds including 34 % Asian, 33 % from two or more races, 18 % White, 14 % Native Hawai‘ian and Pacific Islander, and 10 % Latino.^19^ The median income for a household is $39,139, with 17 % of the population below the poverty line.^19^ Hilo is served by one 276-bed hospital (Hilo Medical Center), one hospice (the Hospice of Hilo), and 30 primary care physicians. During 2013, we implemented our suite of ACP videos in Hilo before commencing similar activities in other counties in subsequent years (Fig. 1). The Institutional Review Board of the University of Hawai‘i approved this study.

### Intervention

The intervention consisted of a single, 1- to 4-h training and access to the ACP video decision aids. The training focused on the use of video decision aids in supporting—not replacing—ACP conversations. The videos were made available on a mobile app and via web-streaming. Training and use of the videos by clinicians began at Hilo Medical Center in the beginning of the first quarter of 2013, was initiated at the Hospice of Hilo during the second quarter of 2013, and then proceeded with primary care providers during the third quarter of 2013 (Fig. 1). We did not dictate when or if providers should show a video, which providers would be involved, which videos to use, if videos were viewed by patients and/or their family or surrogate, or how these interactions would be documented.^20^,^21^

As part of a broader effort to promote ACP, in 2012 HMSA included ACP as one of their Pay-for-Quality measures for all hospitals in the state, a year prior to the videos being implemented in Hilo. In 2014, all primary care providers in the state became eligible for financial incentives for having ACP discussions with HMSA Medicare Advantage and Commercial members (Appendix B, available online).

The videos attempt to provide a general framework in which to understand ACP including the broad questions that patients should reflect upon and how individual preferences can be translated into actionable medical orders and interventions. All the videos are created to minimize health literacy barriers and are less than 10 min long (http://www.ACPdecisions.org/patients/; Appendix A, available online).^5^–^14^

### Outcomes and Data Collection

Hilo Medical Center staff focused on all adult inpatients with late-stage disease at the time of admission for improved rates of ACP documentation (Appendix C, available online). ACP documentation included completion of an advance directive, designating a durable power of attorney, a Physician Orders for Life-Sustaining Treatment (POLST) form, or any documentation of the patient’s ACP preferences in the medical record. Review of the medical records and presence of ACP documentation was performed by the hospital’s Quality Improvement team; no feedback was provided to any providers regarding ACP documentation. Accordingly, our primary outcome in the hospital setting was defined as change in the rate of ACP documentation among patients with late-stage disease admitted at Hilo Medical Center from the 3 months before to 21 months after the introduction of the videos in January 2013. Three months was chosen as the baseline collection because of HMSA’s plans to disseminate the videos in Hilo Medical Center during January of 2013, the 4th month of the project; 21 months was chosen as the intervention phase because of HMSA’s larger strategy to disseminate the videos to other parts of the state at that time.

All 30 primary care providers in Hilo were offered use of the videos. Accordingly, our primary outcome in the outpatient setting compared rates of ACP documentation for all HMSA patients over the age of 75 years in Hilo to rates in the rest of the state during 2014, the first full year of video use in Hilo. Data on outpatient ACP documentation were not available prior to the initiative. We chose 75 as the age cutoff in the outpatient setting since HMSA chose outpatients over the age of 75 to focus on during this initial phase of ACP expansion.

In addition, we evaluated parameters related to hospice utilization in Hilo. Specifically, we measured the number of hospice admissions of late-stage hospitalized patients from the 3 months before to the 21 months after the introduction of the videos in January 2013. This was compared to rates of hospice admissions in neighboring counties (Maui, Kauai, and West Hawai‘i) combined as the Hawai‘i control region and nationally during this same period.^22^,^23^ Maui, Kauai, and West Hawai‘i were used as the control region as they represent all of the comparable healthcare markets in the state. We also used a state database for inpatient deaths of people over 65 to compare the rate of death among hospitalized patients over the age of 65 at Hilo Medical Center versus the state of Hawai‘i control region.

We also examined health plan reimbursement for the last month of life for patients who had been hospitalized within the last 2 months prior to death. We used HMSA claims data from Hilo for the year before (2012) to the year after initiating the intervention (2013) and to similar patients in terms of age and clinical status in the Hawai‘i control region.

### Statistical Analysis

The ACP documentation rates for late-stage patients at Hilo Medical Center and for patients over the age of 75 among primary care providers before and after the intervention were compared with chi-squared and t-tests. The portion of late-stage patients discharged to hospice before and after the intervention were compared with a chi-squared test. The change in the number of hospice admissions was evaluated using a difference-in-differences approach in which changes before and after the intervention were compared between Hilo and a comparison population of similar patients in the Hawai‘i control region and national hospice data using t-tests. The changes in death rate among hospitalized patients over the age of 65 and cost in the last month of life were evaluated using a difference-in-differences^24^ approach in which changes before and after the intervention were compared between Hilo and a comparison population of similar patients in the Hawai‘i control region using t-tests.

## RESULTS

### ACP Documentation

#### Inpatients

A total of 346 adult inpatients with late-stage disease were admitted to Hilo Medical Center during the 3 months before the implementation of the videos and training of providers (Table 1). The average age was 67.0 years (SD = 15.9), and 45.0 % were female. Admission diagnoses included advanced heart failure (17.3 %), advanced chronic obstructive pulmonary disease (15.6 %), cerebrovascular disease (12.4 %), advanced renal disease (9.5 %), advanced cancer (8.7 %), among other diseases.

**Table 1:** Hilo Medical Center Inpatients’ Characteristics

Variables	Baseline control (N = 346)	Video intervention (N = 2,773)	P value
Age, mean (SD), years	67.0 (15.9)	70.2 (15.0)	<0.01
Female, N (%)	156 (45.0)	1,292 (46.6)	0.28
Admitting diagnosis, N (%)			<0.01
Heart failure	60 (17.3)	321 (11.6)	
Cancer	30 (8.7)	320 (11.5)	
Other pulmonary conditions	19 (5.5)	262 (9.4)	
COPD^a^	54 (15.6)	203 (7.3)	
Septicemia	0 (0)	188 (6.8)	
Renal disease	33 (9.5)	135 (4.9)	
Cerebrovascular disease	43 (12.4)	117 (4.2)	
Myocardial infarction	1 (0.3)	98 (3.5)	
Other	106 (30.6)	1129 (40.7)	

^a^ *COPD* Chronic obstructive pulmonary disease

During the 21-month implementation period (first quarter of 2013 through third quarter of 2014), a total of 2,773 adult inpatients with late-stage disease were admitted to Hilo Medical Center (Table 1). The average age was 70.2 years (SD = 15.0), and 46.6 % were female. Admission diagnoses included advanced heart failure (11.6 %), advanced cancer (11.5 %), advanced chronic obstructive pulmonary disease (7.3 %), septicemia (6.8 %), advanced renal disease (4.9 %), cerebrovascular disease (4.2 %), among other diseases.

**Fig. 2:**
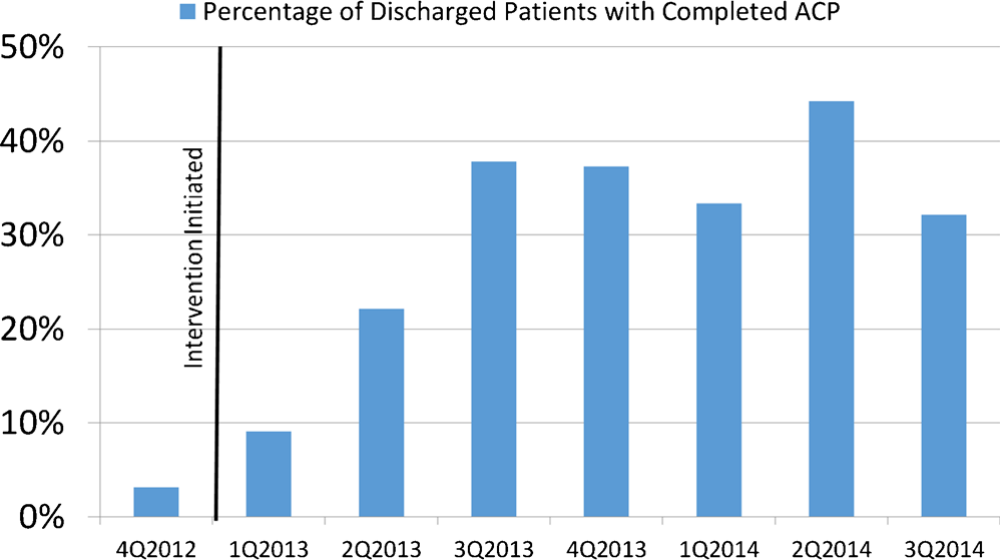
Completion of ACP documents by patients with late-stage disease hospitalized at Hilo Medical Center

Prior to implementation of the videos, the rate of ACP completion for patients with late-stage disease was 3.2 % (11 late-stage patients with ACP/346 late-stage patients admitted in the 3 months prior to intervention). In the 21 months after the intervention, there was an increase in ACP documentation to 39.9 % (1,107 late-stage patients with ACP/2,773 late-stage patients admitted after video intervention) (*P* < 0.001) (Fig. 2).

#### Outpatients

During 2014, 45,987 HMSA patients were aged 75 or older and had a primary care provider in the state of Hawai‘i. The primary care providers in Hilo cared for 3,888 of these patients [average age: 84.0 (SD = 5.8); female: 59 %] (Table 2). The remaining 42,099 patients were cared for by all other providers in the state [average age: 84.1 (SD = 5.9); female: 61 %] (Table 2).

**Table 2:** Characteristics of Outpatients over the Age of 75

Variables	All PCPs not in Hilo (N = 42,099)	All PCPs in Hilo (N = 3,888)	P Value
Age, mean (SD), years	84.1 (5.9)	84.0 (5.8)	1.00
Female, N (%)	25,680 (61.0)	2,294 (59.0)	0.11

^a^ *PCPs* Primary care providers

Primary care providers in Hilo had an ACP completion rate for this cohort of 37.0 % (1,437/3,888) compared to all other providers in the state, who had an average of 25.6 % (10,760/42,099) (*P* < 0.001).

### Hospice

**Fig. 3:**
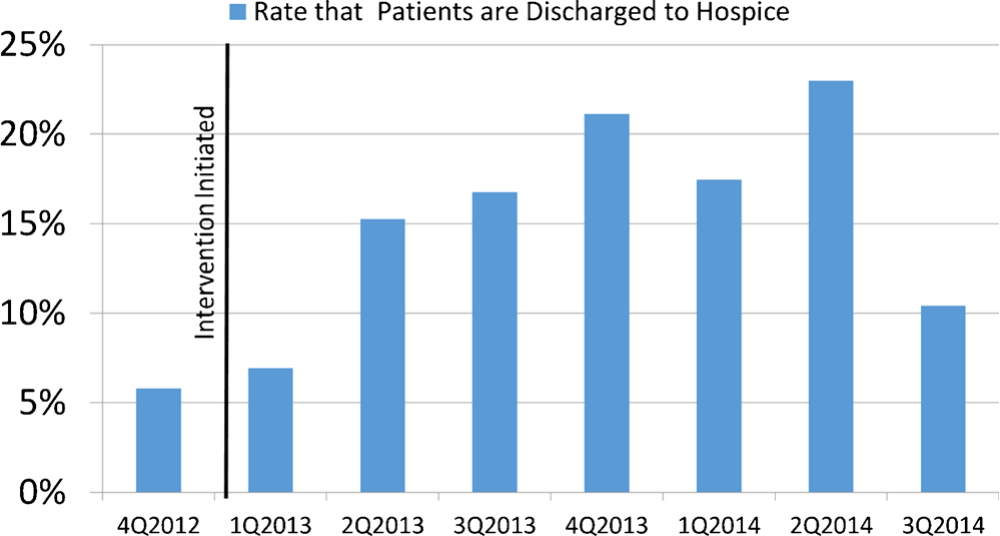
Late-stage patients discharged to hospice from Hilo Medical Center

Prior to implementation of the videos, the rate of discharge to hospice for hospitalized patients with late-stage disease was 5.7 % (20 late-stage hospitalized patients discharged to hospice/346 late-stage patients admitted in the 3 months prior to intervention). After the intervention, there was an increase of late-stage patients discharged to hospice to 13.8 % (382 late-stage patients discharged to hospice/2,773 late-stage patients admitted after video intervention over 21 months of implementation) (*P* < 0.001) (Fig. 3).

Hospice of Hilo admissions grew at a rate of 28.3 % and 50.7 %, respectively, for 2013 and 2014 compared to 2012, which was more than the rates of hospice growth for the Hawai‘i control region (11.7 % and 34.5 %, respectively; *P* < 0.01 for both) and national hospice growth for admissions (3.8 % and 18.7 %, respectively; *P* < 0.01 for both) during the same period.

### Deaths Among Hospitalized Patients over 65

**Fig. 4:**
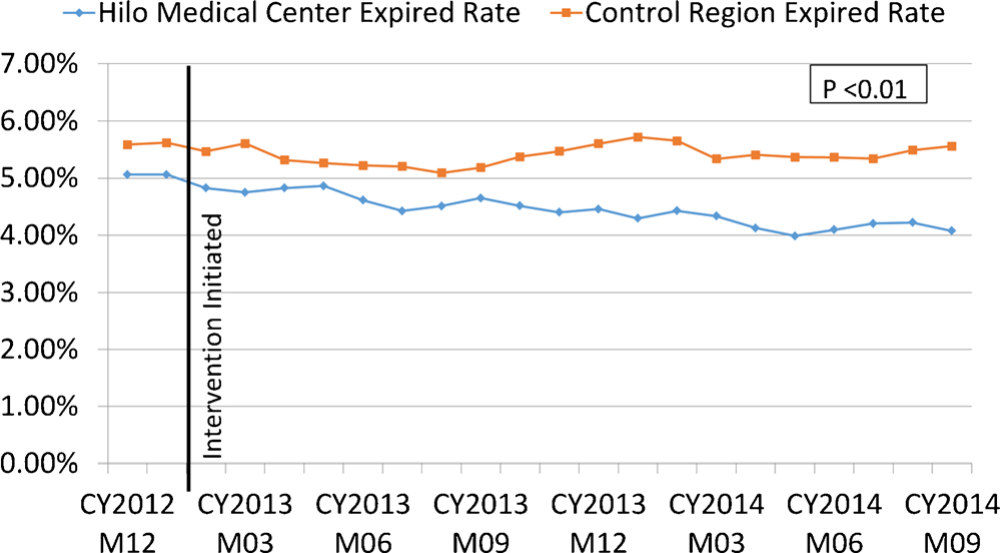
In-hospital death rate for patients over the age of 65, Hilo and control region

There was a decrease in the rate of death in Hilo Medical Center among hospitalized patients over 65 years after the intervention. The hospital death rate for inpatients over 65 in 2012, prior to the intervention, was 5.1 % (145/2,864); during the 21 months after the intervention, the rate decreased to 4.3 % (220/5,068) (*P* = 0.14). For the control region, the hospital death rate for inpatients over 65 in 2012 was 5.6 % (365/6,533) and changed to 5.5 % (653/11,828) during the same 21 months. Figure 4 shows that the monthly rate of death among hospitalized patients over the age of 65 using a rolling 12-month count for patients is lower at Hilo Medical Center than for the Hawai‘i control region (*P* < 0.01). The average monthly difference for the rate of death among hospitalized patients in Hilo versus the control region—minus baseline differences between Hilo and the control region—(i.e., the difference-in-differences) was 0.42 % (95 % confidence interval 0.39 % to 0.45 %).

## COST

The average costs (i.e., health plan reimbursement) for the last month of life increased among patients in both Hilo and the control region between 2012 and 2013. In Hilo, costs were $1,215 (5.5 %) higher in 2013 than in 2012. In the Hawai‘i control region, costs were $4,673 (22.1 %) higher in 2013 than in 2012 (*P* < 0.05). The average total health plan reimbursement for the last month of life in Hilo versus the control region in 2013—minus costs in 2012—(i.e., the difference-in-differences) was $3,458 (95 % confidence interval $3,051 to 3,865) lower per patient.

## DISCUSSION

This article explored the first phase of dissemination of ACP videos in the city of Hilo as part of a larger implementation program throughout the state of Hawai‘i. After the intervention, there was an increase in ACP documentation of patients’ preferences in the inpatient and outpatient settings. In addition, more patients with an advanced illness were likely to be discharged to hospice, which was reflected in decreased hospital death rates. In the year after implementation of the videos, the hospice admission rate grew in Hilo (28 %) relative to the rest of the state (12 %) and national trends (4 %) and decreased costs in the last month of life for decedents.

This study provides important insights. Although ACP has been promoted as a promising avenue to improve the delivery of care at the end of life, scalable and cost-effective methods have not previously been examined. The Centers for Medicare and Medicaid Services’ recent approval to reimburse doctors for having ACP conversations is an important start;^2^ many providers and patients can benefit from using evidence-based tools to get the most out of these encounters. Leveraging decision aids to inform patients and families about their medical options can quickly and inexpensively communicate information.^15^ We have shown in our previous randomized trials that ACP video decision aids can empower and activate patients, providers, and health systems to disrupt healthcare and attempt to deliver medical care to patients that is aligned with their preferences.^5^–^15^ Video decision aids can help ensure that these discussions are high-quality, informed encounters where patients can better understand their medical options, make decisions, and effectively communicate them to someone who can safeguard that they will be honored.^5^–^15^

In this study, the video tools appeared to catalyze ACP documentation, a rate-limiting step in patient-centered care.^25^ We believe that the success of the intervention shows how clinicians and patients can be empowered to engage in ACP and potentially reduce undesired treatment in our healthcare system.^4^ Decision aids can play an important role in prompting discussions with providers who may not feel that they were adequately trained to have these discussions otherwise.

Our study has several important limitations. First, our study was conducted in one of the smallest states in the country, which may limit the generalizability of our findings. This is a common critique of research in Hawai‘i, but the diversity of the state may countervail the influence of the state’s size. Additionally, a significant strength of work in Hawai‘i is that its relative isolation limits the influence of external influences, which may enhance or blunt the impact of interventions. Our study in Hilo allowed for detection on multiple fronts (inpatient, outpatient, community) of a scalable intervention that we show can influence healthcare in a relatively brief span of time.

Second, our study included the first phase of our intervention. Future work will explore the results of the work we have done to extend the intervention to the entire state of Hawai‘i. Today in Hawai‘i, all hospitals, ten hospices, military facilities, and many providers in the state are using the videos. Our work now includes chaplains, religious leaders, social workers, estate lawyers, and others in implementing ACP more broadly as a social and cultural engagement. Future work will explore results of the broader implementation.

Third, during the implementation period there was a pay-for-quality financial incentive for the hospital and primary care providers to improve ACP that may have accounted for some of the improved outcomes. However, this is not likely to be the cause of the differences we report. All the hospitals had the same incentive starting in 2012, a year before the start of our implementation in Hilo. The incentives for primary care providers began in 2014 and were for all providers in the state. Any influence would be present across Hawai‘i. Furthermore, use of financial incentives is common in clinical care and real-world implementations.

Fourth, we did not have a tracking mechanism to identify which videos were shown or how often videos were viewed, i.e., we have no measure of intervention fidelity. Targeted patients not seeing the video represent a likely bias toward the null leading to an underestimate of the intervention effect. We cannot estimate the size of this underestimation.

Finally, we did not require clinicians to show the videos or keep track of who watched the video, but in keeping with the spirit of a real-world trial, allowed them to use the videos as they wished and to the extent that they felt was warranted.

ACP offers the potential to decrease unwanted, invasive, ineffective, and costly medical care at the end of life. Traditional means of training providers and patients on ACP is likely beyond the reach of the vast majority of healthcare systems because of the costs, logistics, and the immediate needs of our most vulnerable and sickest patients. Decision aids are part of a comprehensive solution that can address this misalignment in medical care. Unlike previous interventions that were promoted without evidence,^18^ we provide evidence in both controlled trials and the current implementation study that video decision aids can help align patient preferences with care delivered.

## Electronic supplementary material

Below is the link to the electronic supplementary material.

ESM 1 List of Video Decision Aids. (DOC 41 kb)

ESM 2 Pay-for-Quality measures for their Medicare and commercial members. (DOC 37 kb)

ESM 3 Definition of late-stage disease at Hilo Medical Center. (DOC 32 kb)
