# Supplementary material for: Use of Video Decision Aids to Promote Advance Care Planning in Hilo, Hawai‘i
Source: J Gen Intern Med. 2016 May 18;31(9):1035–40. doi: 10.1007/s11606-016-3730-2 (PMC4978682; doi:10.1007/s11606-016-3730-2)
Supplement: Supplementary file 1 — List of Video Decision Aids. (DOC 41 kb) [file 11606_2016_3730_MOESM1_ESM.doc]

**Appendix A: The Advance Care Planning Video Library (Provider Chose Any Video from the Below).**

**Hawaii Collection**

**(filmed with narrators from Hawaii)**

Goals of Care: A General Overview

Goals of Care: Advanced Disease

CPR: A General Overview

CPR: Advanced Disease

Hospice: An Introduction

Palliative Care: An Introduction

Advance Care Planning for Healthy Adults

**Goals of Care**

Goals of Care: A General Overview

Goals of Care: Advanced Disease

Goals of Care: In the ICU

Goals of Care: Advanced Cancer

Goals of Care: Advanced Heart Failure

Goals of Care: Advanced Dementia

Goals of Care: A General Overview (Cantonese)

Goals of Care: Advanced Disease (Cantonese)

Goals of Care: Advanced Cancer (Cantonese)

Goals of Care: Advanced Heart Failure (Cantonese)

Goals of Care: A General Overview (Ilocano)

Goals of Care: A General Overview (Japanese)

Goals of Care: A General Overview (Korean)

Goals of Care: Advanced Disease (Korean)

Goals of Care: A General Overview (Marshallese)

Goals of Care: A General Overview (Samoan)

Goals of Care: Advanced Disease (Samoan)

Goals of Care: A General Overview (Tagalog)

Goals of Care: A General Overview (Vietnamese)

**CPR**

CPR: A General Overview

CPR: Advanced Disease

CPR: Advanced Cancer

CPR: Advanced Heart Failure

CPR: A General Overview (Cantonese)

CPR: Advanced Cancer (Cantonese)

CPR: Advanced Heart Failure (Cantonese)

**Palliative Care**

What is Palliative Care? (Extended Version)

What is Palliative Care? (Condensed Version)

**Hospice**

Hospice: An Introduction

Hospice: An Introduction in Patients with Advanced Cancer

Hospice: An Introduction (Cantonese)

Hospice: An Introduction in Patients with Advanced Cancer (Cantonese)

Hospice: An Introduction (Ilocano)

Hospice: An Introduction (Japanese)

Hospice: An Introduction (Korean)

Hospice: An Introduction (Tagalog)

Hospice: An Introduction (Vietnamese)

**Advanced Cancer**

Goals of Care: Advanced Cancer^*^

CPR: Advanced Cancer^*^

Hospice: An Introduction in Patients with Advanced Cancer^*^

**Advanced Heart Failure**

Goals of Care: Advanced Heart Failure^*^

CPR: Advanced Heart Failure^*^

**Advanced Dementia**

Goals of Care: Advanced Dementia^*^

Advanced Dementia Overview

Feeding Tubes

**Renal Disease**

Dialysis and Other Options for People 75 & Over with Advanced Kidney Disease

**Informed Consent**

Informed Consent for Procedures in the ICU

**Spanish Collection**

Objetivos del Cuidado: Observaciones Generales

Objetivos del Cuidado: Observaciones sobre Cancer

Decisiones sobre Cuidado de Hospicio: Observaciones Generales

Decisiones sobre Cuidado de Hospicio: Observaciones sobre Cancer

CPR en Español: Cancer Avanzado

CPR en Español: Enfermades Graves

**Healthy Adults Collection**

A General Overview of Advance Care Planning for Healthy Adults (Extended Version)

A General Overview of Advance Care Planning for Healthy Adults (Condensed Version)

How to Talk to Your Doctor About ACP

Advance Directives

**Provider Videos**

How to have The Conversation

Introduction to ACP Videos

How to Implement ACP Videos

^*^ Listed Twice

Description of the videos:

The goals-of-care videos begin with a physician introducing the viewer to ACP and a three category goals-of-care framework (life-prolonging care, limited medical care, and comfort care), which we have previously tested. The images in each category reflect the interventions primarily involved. For life-prolonging care, the images include a simulated code with a mannequin as well as an intensive care unit with a ventilated patient being tended by respiratory therapists. Visual images depicting limited medical care include a patient getting medications via a peripheral intravenous catheter and scenes from a typical medical ward service with a patient wearing a nasal cannula. The video depiction of comfort care includes a patient receiving oral medications at home and a medical attendant assisting a patient with self-care. Additional videos explore other important ACP decision points: hospice, palliative care, feeding tubes, and ACP more generally.

In its development, the videos’ design, script, scenes, and structure were reviewed for accuracy and appropriateness by experts in all the major medical disciplines with a focus on geriatrics, palliative care, decision-making, health literacy, and medical ethics. The VIDEO team performed all filming and editing of the videos following previously published criteria. All providers and patients included in the videos (or their proxies) gave consent to be filmed. Translation and cultural adaptation of the videos underwent a similar review process and all videos are narrated by a native speaker.

A sample of the videos can be viewed at <http://www.ACPdecisions.org/patients/>
