# Supplementary material for: Use of Video Decision Aids to Promote Advance Care Planning in Hilo, Hawai‘i
Source: J Gen Intern Med. 2016 May 18;31(9):1035–40. doi: 10.1007/s11606-016-3730-2 (PMC4978682; doi:10.1007/s11606-016-3730-2)
Supplement: Supplementary file 2 — Pay-for-Quality measures for their Medicare and commercial members. (DOC 37 kb) [file 11606_2016_3730_MOESM2_ESM.doc]

**Appendix B**

**HMSA’s Advanced Hospital Care program:**

**Advanced Care Planning (ACP) Initiatives**

Since 2012, ACP has had a significant role in HMSA’s Advanced Hospital Care Program. In the first year of the program, HMSA supported implementation of ACP by incentivizing hospitals to develop a systematic approach for patients nearing end of life, as well as provide staff education for conducting ACP discussions and documenting patients’ health care choices in the medical record. HMSA emphasizes the need for developing ACP, including the capacity to deliver services to patients approaching end of life by:

- Documenting patients’ preferences for the care they receive during end of life.
- Ensuring that caregivers have access to such documentation.

Program goals include:

1. Identifying appropriate patients at high risk for death during or within a year of hospitalization;
2. Initiating ACP before a medical crisis for appropriately identified patients;
3. Assisting patients in creating an individualized plan;

**2012 – 2014 Requirements**

1. Develop a document that describes hospital current capability to deliver end-of-life services including personnel, training programs, processes in place, and data pertinent to this topic.
2. Develop and implement an algorithm to identify patients with life limiting prognosis diagnoses, who might benefit from ACP. ACP is defined as any education/consultation and palliative care intervention.
3. Develop a policy and procedures to identify the targeted cohort.
4. Identify staff and physician resources to intervene with the identified cohort.
5. Develop education standards and educate ACP Planning staff.
6. Track outcomes for the end-of-life cohort on the following metrics:
   - Number of identified patients with a documented ACP intervention of any type.
   - Number of identified patients with a documented ACP consultation.
   - Hospice referrals.

# Advance Care Planning Primary Care Pay for Quality Measure

## Description

The percentage of patients 75 years of age and older at the end of the measurement period who had an advance care plan and/or an advance care planning discussion with their PCP documented during the measurement period.

**Numerator**

Advance care discussion or plan documented in the medical record and a CPT code submitted during the measurement period. Acceptable Documentation include:

1. Advance care plan documents
   1. Durable power of attorney for health care decision-making.
   2. Executed advance health care directive.
   3. Physician orders for life-sustaining treatment (POLST).
2. Advance care plan discussion: If your patient is uncomfortable or not ready to create an advance care plan document, the patient’s chart should document the patient’s current thinking about at least one of the following:
   1. CPR.
   2. Goals of care for cardiopulmonary failure, including hospitalization.
   3. Artificial nutrition and hydration.
   4. Comfort care option.
   5. Advance directive decisions.
   6. Durable power of attorney for health care/designated surrogate.
   7. The patient’s key questions for further discussion.
   8. The progression of their illness.
   9. Potential complications.
   10. Specific life-sustaining treatments that may be required if their illness progresses.

Providers can submit either through a CPT code submitted during the measurement period or supplemental data with documentation upload. The following codes identify advance care planning:

CODES

- CPT-II Codes 1157F–Advance care plan or similar legal document present in the medical record
- CPT-II Codes 1158F–Advance care plan discussion in the medical record
- HCPCS S0257–Counseling and discussion regarding advance directives or end-of-life

care planning and decisions, with patient and/or surrogate (list separately in addition to code for appropriate evaluation and management service)

- ICD-9-CM Diagnosis V49.86–Do not resuscitate status
