# Supplementary material for: Use of Video Decision Aids to Promote Advance Care Planning in Hilo, Hawai‘i
Source: J Gen Intern Med. 2016 May 18;31(9):1035–40. doi: 10.1007/s11606-016-3730-2 (PMC4978682; doi:10.1007/s11606-016-3730-2)
Supplement: Supplementary file 3 — Definition of late-stage disease at Hilo Medical Center. (DOC 32 kb) [file 11606_2016_3730_MOESM3_ESM.doc]

**Appendix C: Definition of Late-Stage Disease at Hilo Medical Center**

Late-stage disease was defined as a patient with any of the following admitting diagnoses: advanced heart failure (CHF), cerebrovascular disease, advanced lung disease (COPD), advanced dementia, end-stage renal disease, cancer, HIV/AIDS, advanced diabetes with complications (DM), frailty, or failure to thrive. The admitting attending determined whether or not the patient had one or more of the above admitting diagnoses for late-stage disease and whether the patient was appropriate for viewing a video.
